# Exploring Differences in Speech Processing Among Older Hearing-Impaired Listeners With or Without Hearing Aid Experience: Eye-Tracking and fMRI Measurements

**DOI:** 10.3389/fnins.2019.00420

**Published:** 2019-05-03

**Authors:** Julia Habicht, Oliver Behler, Birger Kollmeier, Tobias Neher

**Affiliations:** ^1^Medizinische Physik and Cluster of Excellence “Hearing4all”, Oldenburg University, Oldenburg, Germany; ^2^Institute of Clinical Research, University of Southern Denmark, Odense, Denmark

**Keywords:** hearing loss, hearing aids, plasticity, speech comprehension, eye-tracking, fMRI

## Abstract

Recently, evidence has been accumulating that untreated hearing loss can lead to neurophysiological changes that affect speech processing abilities in noise. To shed more light on how aiding may impact these effects, this study explored the influence of hearing aid (HA) experience on the cognitive processes underlying speech comprehension. Eye-tracking and functional magnetic resonance imaging (fMRI) measurements were carried out with acoustic sentence-in-noise (SiN) stimuli complemented by pairs of pictures that either correctly (target picture) or incorrectly (competitor picture) depicted the sentence meanings. For the eye-tracking measurements, the time taken by the participants to start fixating the target picture (the ‘processing time’) was measured. For the fMRI measurements, brain activation inferred from blood-oxygen-level dependent responses following sentence comprehension was measured. A noise-only condition was also included. Groups of older hearing-impaired individuals matched in terms of age, hearing loss, and working memory capacity with (eHA; *N* = 13) or without (iHA; *N* = 14) HA experience participated. All acoustic stimuli were presented via earphones with individual linear amplification to ensure audibility. Consistent with previous findings, the iHA group had significantly longer (poorer) processing times than the eHA group, despite no differences in speech recognition performance. Concerning the fMRI measurements, there were indications of less brain activation in some right frontal areas for SiN relative to noise-only stimuli in the eHA group compared to the iHA group. Together, these results suggest that HA experience leads to faster speech-in-noise processing, possibly related to less recruitment of brain regions outside the core sentence-comprehension network. Follow-up research is needed to substantiate the findings related to changes in cortical speech processing with HA use.

## Introduction

Age-related hearing loss is a common chronic health condition that often remains untreated. Despite substantial advancements in hearing aid (HA) technology over the last decades, less than 25% of the hearing-impaired population over the age of 60 use HAs (e.g., Popelka et al., 1998; Lin et al., 2011; Feder et al., 2015). Recently, evidence has been accumulating that untreated hearing loss can lead to declines in brain volume and compensatory changes in cortical resource allocation with important consequences for communication abilities (Wong et al., 2010; Peelle et al., 2011; Eckert et al., 2012; Lin et al., 2014). Concerning the influence of hearing device treatment, there is evidence for rapid adaptation in auditory cortex following cochlear implantation. For instance, a recent longitudinal study based on electroencephalography found increased amplitudes and shorter latencies of the N1 wave after 8 weeks of cochlear implant use (Sandmann et al., 2015). Regarding HA provision, findings concerning cortical adaptation are inconsistent. To illustrate, Dawes et al. (2014) fitted groups of novice and experienced HA users with bilateral HAs and performed event-related potential measurements before and after 12 weeks of HA use. Focusing on the N1 and P2 components, these authors did not observe any changes as a result of HA use. In contrast, Hwang et al. (2006) fitted bilaterally hearing-impaired participants with unilateral HAs and measured blood-oxygen-level dependent (BOLD) responses using functional magnetic resonance imaging (fMRI). After 3 months of HA use, their participants showed a bilateral decrease in brain activation in auditory cortex (i.e., superior temporal gyrus).

A possible explanation for the inconsistent HA results could be that they were obtained using different experimental paradigms. In a series of previous experiments, we used an eye-tracking paradigm developed by Wendt et al. (2014) to investigate the effects of HA experience on how quickly a participant can grasp the *meaning* of acoustic sentence-in-noise (SiN) stimuli presented together with two similar pictures that either correctly (target picture) or incorrectly (distractor picture) depict the sentence meanings. The participant’s task is to identify the target picture by pressing one of two buttons after sentence presentation. The time taken for this to occur is called the “response time.” Additionally, the time taken by the participant to start fixating the target picture *during* sentence presentation is determined using an eye-tracker. This measure is referred to as the “processing time.” In a cross-sectional study, we compared experienced and inexperienced HA users matched in terms of age, pure-tone average hearing loss (PTA), and working memory capacity in terms of their response and processing times (Habicht et al., 2016). We found longer processing times for the inexperienced HA users than for the experienced HA users, despite comparable stimulus audibility and speech recognition performance. In contrast, the two groups did not differ in their response times. In a longitudinal follow-up study, we acclimatized novice users to bilateral HA fittings (Habicht et al., 2018). Before acclimatization, these participants had significantly longer processing times than a control group of experienced HA users. After 24 weeks of HA use, the processing (but not response) times of the novice users improved by almost 30%, thereby reaching the level of the controls. Together, these findings imply that HA use leads to faster speech comprehension in noise as measured using processing times.

Neurophysiological research has shown that speech comprehension relies on a core frontotemporal sentence-comprehension network of brain regions that includes bilateral temporal cortex (superior temporal sulcus, middle temporal gyrus), left inferior frontal gyrus and left precentral gyrus, and (pre-) supplementary motor area (e.g., Adank, 2012; Lee et al., 2016). The activated cortical areas appear to change with the task requirements, age, and hearing loss. For example, deciphering the meaning of sentences with different linguistic complexities leads to the recruitment of different brain regions, and the observable activation patterns vary also with age. To illustrate, Peelle et al. (2009) presented sentences with low or high linguistic complexity to young and older normal-hearing adults and measured BOLD responses following sentence comprehension. The participants had to press one of two buttons to identify the gender of the character performing the action in a given sentence. Both groups showed stronger activation in left inferior and middle frontal gyri, left inferior parietal cortex, and left middle temporal gyrus for high- compared to low-complexity sentences. Furthermore, relative to the younger group the older group had decreased activation in inferior frontal regions but increased activation in frontal regions outside the core sentence-comprehension network. Concerning the influence of hearing loss, Peelle et al. (2011) tested older adults with high-frequency audiometric hearing thresholds ranging from clinically normal to moderately elevated. They observed a negative correlation between hearing thresholds and brain activation to sentence stimuli in bilateral superior temporal gyri (including auditory cortex), thalamus, and brainstem.

The findings summarized above are consistent with the idea that both age and hearing loss lead to the recruitment of cortical areas outside the core sentence-comprehension network, possibly as a result of compensatory mechanisms for achieving speech comprehension. Because previous research did not consider the effects of HA treatment on cortical speech processing, it is currently unclear if experience with amplified sound plays a role for this. The purpose of the current study was to address this knowledge gap. First, to confirm the previously observed difference in processing times and to provide a baseline for the other measurements, we tested experienced and inexperienced HA users using the eye-tracking paradigm described above. We then performed fMRI measurements to explore differences in brain activation during speech comprehension among the two participant groups. For that purpose, we adapted the eye-tracking paradigm for BOLD response measurements. In addition, we included a noise-only condition as a reference. Based on our earlier eye-tracking results, we expected longer processing times for inexperienced compared to experienced HA users. Additionally, based on the fMRI literature summarized above, we hypothesized that our inexperienced users would show more brain activation to SiN stimuli outside the core frontotemporal sentence-comprehension network.

## Materials and Methods

### Participants

**FIGURE 1:**
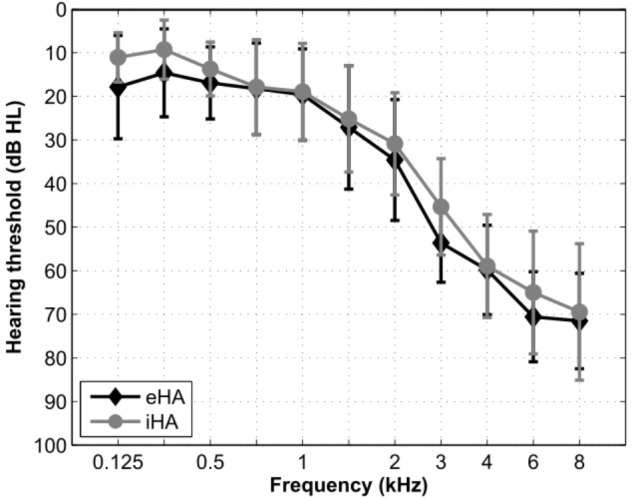
Mean hearing threshold levels across the left and right ears of the eHA and iHA participants.

A total of 27 right-handed participants were recruited from a large database of hearing-impaired listeners available at the Hörzentrum Oldenburg GmbH. Thirteen of the participants were experienced HA users with at least 1 year (mean: 4.5 years; *SD*: 2.7 years) of bilateral HA experience (eHA). The other 14 participants were inexperienced HA users with no previous HA experience (iHA). Their motivation for participating in the current study was primarily due to them being able to try out HAs without any subsequent commitments. Inclusion criteria for all of these participants were (1) age between 60 and 80 years, (2) bilateral, sloping, sensorineural hearing loss in the range from 40 to 80 dB HL between 3 and 8 kHz, (3) self-reported normal or corrected-to-normal vision, and (4) no conditions that would be contraindicative for fMRI measurements (e.g., a pacemaker). One inexperienced subject was excluded from the fMRI analysis due to structural abnormalities identified in the anatomical scan. Table 1 summarizes the main characteristics of the two groups of participants. Figure 1 shows mean hearing threshold levels. Pure-tone average hearing loss as calculated across ears for the four standard audiometric frequencies from 0.5 to 4 kHz (PTA4) was 34 and 31 dB HL for the eHA and iHA group, respectively. Two independent *t*-tests showed no group differences in terms of age (*t*_25_ = -0.01, *p* > 0.5) or PTA4 (*t*_25_ = 1.0, *p* > 0.05).

**Table 1:** Means (and standard deviations) for age, PTA4, reading span, and SRT80s for the two levels of linguistic complexity (low, high) and participant groups.

	eHA	iHA
***N***	**13**	**13**

Age (years)	68.8	68.4
	(4.04)	(5.69)
PTA4 (dB HL)	33.9	31.7
	(7.41)	(6.96)
Reading span (%-correct)	43.0	38.0
	(11.8)	(14.4)
SRT80_low_ (dB SNR)	-1.9	-2.1
	(0.8)	(1.0)
SRT80_high_ (dB SNR)	-1.2	-1.3
	(1.1)	(0.7)


### Apparatus

With the exception of the fMRI measurements, all measurements took place in a soundproof booth and were performed with a computer equipped with an RME Fireface UCX (Audio AG, Haimhausen, Germany) soundcard. For the eye-tracking measurements, the setup of Wendt et al. (2014) was used (EyeLink 1000 desktop system, EyeLink CL high-speed camera, SR Research, Ltd., Samsung 2253BW monitor). The visual stimuli were presented on a 22^′′^ multiscan computer monitor with a resolution of 1680 × 1050 pixels. The participants were seated such that their eyes were 60 cm in front of the monitor. Using a nine-point fixation stimulus procedure developed by the manufacturer of the eye-tracker, the setup was calibrated at the start of each block of measurements. Before each trial, the participants had to fixate a point at the center of the monitor for drift correction. The behavioral responses (i.e., button presses) of the participants were collected using a computer keyboard. All acoustic stimuli were presented via free-field equalized Sennheiser (Wennebostel, Germany) HDA200 headphones.

For the fMRI measurements, a 3 Tesla MRI system (Siemens Magnetom Prisma 3T, Siemens AG, Erlangen, Germany) with a 32-channel head coil was used. Stimulus presentation and behavioral data collection was carried out using the Cogent 2000 toolbox (Wellcome Department of Imaging Neuroscience) in MATLAB, 2014a (Version 2014a, The MathWorks, Inc., Natick, MA, United States). The visual stimuli were presented via a mirror construction from a projector screen at the end of the tube with a resolution of 1280 × 1024 pixels. An fMRI-compatible button pad (LXPAD-2x5-10M, NAtA Technologies, Coquitlam, BC, Canada) was used for collecting the behavioral responses of the participants. The acoustic stimuli were presented via free-field equalized fMRI-compatible headphones (OptoACTIVE, Optoacoustics, Ltd., Or Yehuda, Israel).

All acoustic stimuli were calibrated with a Brüel & Kjær (B&K; Nærum, Denmark) 4153 artificial ear, a B&K 4134 ½″ microphone, a B&K 2669 preamplifier, and a B&K 2610 measurement amplifier. To ensure audibility, individual linear amplification according to the “National Acoustic Laboratories-Revised” (NAL-R) prescription formula (Byrne and Dillon, 1986) was applied using the Master Hearing Aid research platform (Grimm et al., 2006). For all measurements, stationary speech-shaped noise at a nominal sound pressure level (SPL) of 65 dB was used. The level of the speech signal was individually set in accordance with speech reception threshold (SRT) measurements corresponding to 80%-correct performance (SRT80; see below).

### Working Memory Capacity Measurements

To characterize the participants in terms of their cognitive function, we administered the German reading span test implementation of Carroll et al. (2015) to them. The reading span test is a measure of visual working memory capacity that is rather widely used in cognitive hearing research (e.g., Lunner, 2003; Neher et al., 2014) and that includes three sub-tasks. First, the participant has to read aloud sentence segments displayed successively on a screen and to answer “yes” if the three previous segments made up a meaningful sentence (e.g., “Das Mädchen–sang–ein Lied”; “The girl–sang–a song”) or “no” if they did not make up a meaningful sentence (e.g., “Die Flasche–trank–Wasser”; “The bottle–drank–water”). After a block of three to six sentences, the participant has to repeat as many of the first or final words of the last block of sentences as possible in any order. Altogether, there were three training and 54 test sentences. As the performance measure, we used the percentage of correctly recalled target words across the 54 test sentences.

### Sentence-in-Noise (SiN) Stimuli

For the acoustic stimuli, we used the “Oldenburg corpus of Linguistically and Audiologically Controlled Sentences” (OLACS; Uslar et al., 2013) as speech material. The OLACS consists of seven grammatically correct sentence structures that vary in linguistic complexity. For our measurements, we used two sentence structures: (1) subject-verb-object sentences with a canonical word order and therefore ‘low’ linguistic complexity, and (2) object-verb-subject sentences with a non-canonical word order and therefore ‘high’ linguistic complexity (see Table 2). In each sentence, there are two characters (e.g., a dragon and a panda), one of which (the subject) performs a given action with the other (the object). In the German language, the linguistic complexity of these sentences is determined by relatively subtle grammatical or acoustic cues, e.g., “De***r*** müd***e*** Drach***e*** fesselt de***n*** große***n*** Panda” (meaning: “The tired dragon ties up the big panda”; low complexity) vs. “De***n*** müde***n*** Drach***en*** fesselt de***r*** groß***e*** Panda” (meaning: “The big panda ties up the tired dragon”; high complexity). For the masker signal, we used stationary speech-shaped noise. The noise started 200 ms before and ended 200 ms after the speech signal.

**Table 2:** Example sentences from the “Oldenburg corpus of Linguistically and Audiologically Controlled Sentences” (Uslar et al., 2013) with two levels of linguistic complexity (low, high).

Low	De*r*_nom_	müd*e*_nom_	Drach*e*	fesselt	de*n*_acc_	groß*en*_acc_	Panda.
*Meaning: “The tired dragon ties up the big panda.”*							

**High**	**De*n*_acc_**	**müd*en*_acc_**	**Drach*en***	**fesselt**	**de*r*_nom_**	**groß*e*_nom_**	**Panda.**

*Meaning: “The big panda ties up the tired dragon.”*							


*In each case, the grammatically salient *word endings* (in italics) and corresponding cases (nom, nominative; acc, accusative) are indicated, as are the meanings in English.*

### Visual Stimuli

As visual stimuli, we used the picture sets developed by Wendt et al. (2014) that complement the sentences of the OLACS material. Each picture set consists of two similar pictures that are displayed next to each other and that are presented together with the corresponding acoustic sentence. One of the pictures (the target picture) correctly depicts the meaning conveyed by a given sentence. The other picture (the competitor picture) depicts the same situation but with interchanged roles of the subject and object. For each sentence, there are two picture sets. In one picture set, the left picture is the target; in the other picture set, the right picture is the target.

### Speech Recognition Measurements

Prior to the eye-tracking and fMRI measurements, we assessed baseline speech recognition performance using the OLACS sentences. The task of the participants was to repeat the words they had understood, which an experimenter then scored. Initially, a training measurement based on 20 low-complexity and 20 high-complexity sentences was performed to familiarize the participants with the sentences and the procedure. Using 20 additional sentences per sentence type, we then estimated the SNR corresponding to the SRT80 for the sentences with low or high linguistic complexity.

### Eye-Tracking Measurements

#### Response and Processing Times

**FIGURE 2:**
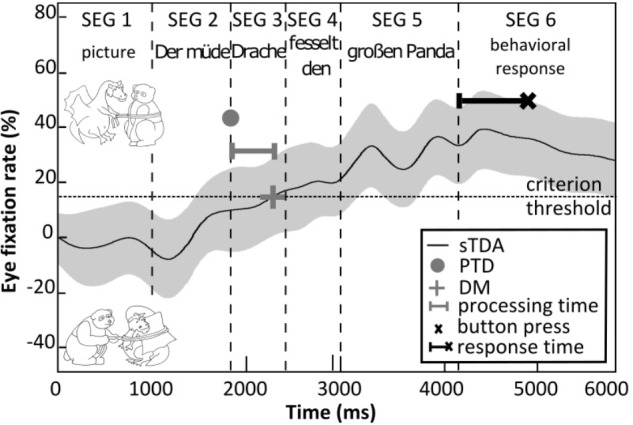
Illustration of the processing time and response time measurements. Shown is the hypothetical eye-fixation rate (sTDA, black line) for an example sentence (“Der müde Drache fesselt den großen Panda” – “The tired dragon ties up the big panda”) with the corresponding picture set (top: target; bottom: distractor) over the course of the acoustic and visual stimulus presentation (segments 1–6). The shaded area illustrates 95% confidence intervals. The gray dot denotes the point of target disambiguation (PTD), which defines the onset of the first word that allows matching the acoustic sentence to the target picture (upper picture). The gray **+** symbol denotes the decision moment (DM), where the eye-fixation rate exceeds the criterion threshold (42%-point of the sTDA maximum, dashed line). The horizontal gray bar corresponds to the processing time, i.e., the time difference between PTD and DM. The black **×** symbol denotes the time point of the button press and the black bar corresponds to the response time.

During the eye-tracking measurements, the acoustic and visual stimuli were presented as illustrated in Figure 2. Initially, the visual stimulus was presented on its own for 1 s (stimulus segment 1) and then the acoustic SiN stimulus was added (stimulus segments 2–5). The participants had to identify the target picture by pressing one of three buttons on the keyboard as quickly as possible after the presentation of the acoustic stimulus (stimulus segment 6): a left button if the target picture appeared to be on the left-hand side, a right button if the target picture appeared to be on the right-hand side, or another button if neither picture appeared to match the spoken sentence. On each trial, the time taken to press the button relative to the end of the spoken sentence was recorded (the ‘response time’). To estimate the processing time, we followed the procedure of Wendt et al. (2015). That is, we determined the eye fixations toward three regions of interest on the computer monitor: (1) the target picture, (2) the competitor picture, and (3) other regions. Based on the recorded eye-fixation data, we first determined the so-called single target detection amplitude (sTDA) for each participant across all sentences of a given level of linguistic complexity. The sTDA is a quantitative, normalized measure across time of the eye-fixation rate of a participant toward the target picture in relation to the eye-fixation rate toward the competitor picture or other regions. Figure 2 shows an example sTDA as a function of time for a sentence with low linguistic complexity. If at a given point in time the target picture was fixated more than the competitor picture (or any other region on the screen), the sTDA is positive. If the competitor picture was fixated more, the sTDA is negative. Since the sentence recordings differed in terms of their durations, the eye fixations were time-aligned by segmenting them in a consistent fashion (Wendt et al., 2014). The processing time was estimated on the basis of the ‘point of target disambiguation’ and the ‘decision moment.’ The point of target disambiguation corresponds to the onset of the first word that allows for disambiguation to occur, i.e., the moment at which the target picture can in principle be identified. For the sentences used here, the point of target disambiguation always corresponded to the start of stimulus segment 3. The decision moment was defined as a relative criterion threshold corresponding to the 42%-point of the sTDA maximum of each test condition (for details see Habicht et al., 2016; Müller et al., 2016). The processing times were then estimated by taking the difference (in milliseconds) between the points of target disambiguation and decision moments. Using this approach, we estimated the processing time for each participant and level of linguistic complexity.

#### Test Blocks

The eye-tracking data were recorded in four blocks. Each block included 37 trials. Specifically, there were 30 trials with 15 low-complexity and 15 high-complexity sentences plus seven catch trials. The catch trials were included to force the participants to look at both pictures and to prevent them from developing other task-solving strategies. We used two types of catch trials. For the first type, we presented either the target or the competitor picture on both sides of the screen. Hence, either both or neither picture matched the spoken sentence. For the second type, we included two additional OLACS sentence types with different grammatical structures (and thus levels of linguistic complexity): subject-relative clauses (e.g., “Der Lehrer, der die Models bestiehlt, zittert” – “The teacher who is stealing from the models is shivering”) and object-relative clauses (e.g., “Der Maler, den die Vampire beschatten, gähnt” – “The painter whom the vampires are shadowing is yawning”). These types of sentences were meant to prevent the participants from getting accustomed to specific sentence structures, thereby forcing them to attend to them continuously. The order of the test blocks was randomized across participants. One test block took about 7 min to complete. In total, each participant carried out 148 test trials (37 trials × 4 test blocks).

#### Statistical Analysis

Initially, we assessed the ability of our participants to identify the target picture by determining the *picture* recognition rate for each level of linguistic complexity. Although the picture recognition rate cannot be directly compared with the SRT80, one would expect picture recognition rates of around 80% and more due to the availability of both acoustic and visual information. On average, the eHA and iHA groups achieved 91.0%-correct (range: 76–98%-correct; *SD*: 0.07%-correct) and 89.5%-correct (range: 72–98%-correct; *SD*: 0.08%-correct) picture recognition rates.

Since the response times were logarithmically distributed, we applied a logarithmic transformation to them. We then used Kolmogorov and Levene’s tests to check the assumptions of normality and homogeneity of variance for our datasets. The transformed response times fulfilled these assumptions (all *p* > 0.05). The same was true for the (non-transformed) processing times (all *p* > 0.05). Thus, we used parametric statistics for analyzing the log response times and processing times further.

### fMRI Measurements

#### Paradigm and Conditions

**FIGURE 3:**
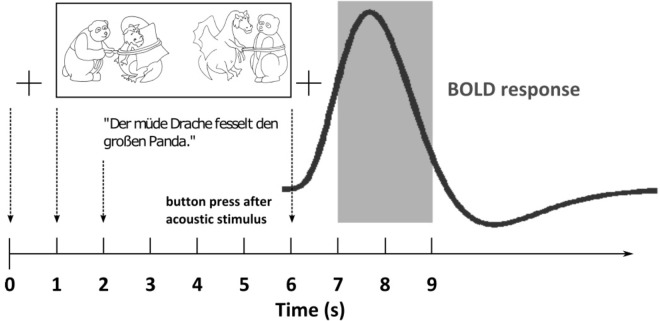
Illustration of the time course of a trial in the functional magnetic resonance imaging (fMRI) paradigm. Shown is the hypothetical BOLD response (thick dark gray line) to an example sentence (“Der müde Drache fesselt den großen Panda” – “The tired dragon ties up the big panda”) with the corresponding picture set over the course of the combined acoustic and visual stimulus presentation. A fixation cross (+) is presented on the screen before and after the stimulus presentation. The gray area denotes the scanner recording.

For the fMRI measurements, we used a sparse temporal sampling strategy to reduce the influence of the acoustic scanner noise (Edmister et al., 1999; Hall et al., 1999) and also modified the eye-tracking paradigm (Figure 3). Similar to the eye-tracking measurements, the visual stimulus was first presented on its own. After 1 s the acoustic stimulus was added. The participants had to identify the target picture by pressing one of two buttons (left or right) on a response pad with their index or middle finger after the presentation of the acoustic stimulus. After 5 s relative to the acoustic stimulus onset, the brain volume was measured, resulting in audible scanner noise for 2 s. Before and after the visual stimulus, a small cross was presented at the center of the screen, which the participants were asked to fixate. We used the same SiN stimuli with the two levels of linguistic complexity (SiN_low_, SiN_high_) together with the corresponding picture sets as used for the eye-tracking measurements. In addition, we included a noise-only condition (i.e., stationary speech-shaped noise) as a reference condition to allow for the detection of speech-specific brain activation in the other conditions. In the noise-only condition, we only displayed one picture of a given picture set during the stimulus presentation. The location of the picture on the screen (left or right) was randomized, and the task of the participant was to identify the location of the picture by pressing the corresponding button on the button pad.

For each participant, we recorded functional fMRI data in one block of 150 trials. Specifically, there were 50 trials per condition (SiN_low_, SiN_high_, noise only). The trials for the three stimulus conditions were presented in randomized order. After completion of the 150 trials, structural images were acquired that served as anatomical references. The fMRI measurements took about 28 min to complete. They were performed at least 3 months after the eye-tracking-measurements, so the participants could not remember the stimuli from the earlier test sessions (Ross and Tremblay, 2009).

#### Data Acquisition

Functional images were obtained using T_2_^∗^-weighted gradient echo planar imaging. Each volume consisted of 34 transversal slices recorded in ascending interleaved order [voxel size 2.2 mm × 2.2 mm× 3 mm, distance factor 20%, repetition time (TR) = 9 s, echo time (TE) = 30 ms, flip angle (FA) = 90°, field of view (FoV) = 204 mm × 204 mm × 122 mm, volume acquisition time = 2 s]. High-resolution structural images were acquired using a T_1_-weighted magnetization-prepared rapid acquisition gradient echo sequence (voxel size 0.7 mm × 0.7 mm × 0.9 mm, distance factor 50%, TR = 2 s, TE = 2.41 ms, FA = 9°, FoV = 230 mm × 194 mm × 187 mm).

#### Preprocessing and General Linear Model

We preprocessed and analyzed the imaging data using SPM 8 (FIL, Wellcome Trust Centre for Neuroimaging, University College London, London, United Kingdom^1^) and MATLAB, r2011b (The MathWorks, Natick, MA, United States). Functional images were realigned to the first image, co-registered to each participant’s structural image, normalized to Montreal Neurological Institute (MNI) space using transformations based on information from structural tissue segmentation (tissue probability maps derived from the structural images), and smoothed with an isotropic Gaussian kernel of 10 mm full-width at half maximum (Peelle et al., 2011).

For each participant, a general linear model was estimated, modeling the signal across time in every voxel of the brain. Each model contained one regressor per stimulus condition. For the SiN_low_ and SiN_high_ conditions, only trials with correct responses were included. All trials corresponding to incorrect responses were modeled as a separate regressor. The six head movement parameters (rigid body translations and rotations) as well as the mean CSF signal^2^ were added as nuisance regressors. Lastly, trials with exceptionally large (and putatively artifactual) changes in the global signal were ‘scrubbed’ by including one additional regressor for each respective spike^3^. Stimulus events were modeled with boxcar functions. A high-pass filter with a cut-off of 1/128 Hz was applied before parameter estimation to remove slow signal drifts, and residual serial correlations in the fMRI time series were accounted for by means of a first-order autoregressive model.

#### BOLD Contrasts

To validate our experimental approach, we investigated the main effects of stimulus type and linguistic complexity on neural activation across subjects by means of statistical parametric maps of second-level random-effects models. At the first level, we generated two sets of contrast images per participant. With the first set, general speech-induced responses were assessed by contrasting all SiN trials (i.e., SiN_low_ and SiN_high_) against all noise trials (SiN > noise). With the second set, we assessed the main effect of linguistic complexity by contrasting the SiN_high_ and SiN_low_ trials (SiN_high_ > SiN_low_). We then entered all resulting first-level contrast images into separate one-sample *t*-tests across all subjects at the second-level. For the purpose of descriptive anatomical localization, we overlaid statistical parametric maps onto the group mean structural image.

To assess the influence of participant group (iHA, eHA) on brain activation related to speech presence and linguistic complexity, we performed two-sample *t*-tests at the second-level. More specifically, we compared the mean activation levels for the SiN > noise contrast as well as the SiN_high_ > SiN_low_ contrast between both groups using one-tailed test statistics (e.g., iHA > eHA for SiN > noise).

We thresholded the resulting whole group *t*-statistic map for the contrast SiN > noise at a significance level of *p* < 0.05, corrected for the family-wise error (FWE) rate. For all other activation maps, this threshold criterion proved too stringent to reveal differences in brain activation patterns. Hence, we used an uncorrected significance level of *p* < 0.001 to explore possible effects of linguistic complexity and HA experience. Additionally, we applied a cluster-extent threshold of 10 voxels to all activation maps. This comparatively lenient approach of thresholding comes along with a higher risk of false positives. On the other hand, it reduces the risk of missing out on real (and potentially important) effects, as for example argued by Lieberman and Cunningham (2009).

Structures corresponding to peaks of significant clusters were determined by means of the Wake Forest University (WFU) Pick atlas^4^ (Maldjian et al., 2003, 2004), using the Talairach Daemon (TD), AAL (Tzourio-Mazoyer et al., 2002), and ICBM labels databases.

### Test Protocol

All participants attended three visits. At the first visit, the SRT80 measurements were carried out. In addition, event-related potential measurements were performed for another study (not shown here). At the second visit, the eye-tracking and working memory capacity measurements were carried out, while at the third visit the fMRI measurements took place. The first and second visit took 2 h each, while the third visit took 1 h.

## Results

### Working Memory Capacity

The results of the reading span test are summarized in Table 1. On average, the eHA and iHA groups could recall 43.0% (*SD*: 12%) and 38.9% (*SD*: 14%) of all target words, respectively. These results are in good agreement with other comparable studies (e.g., Neher et al., 2014; Carroll et al., 2015; Souza et al., 2015). An independent *t*-test revealed no significant difference in terms of reading span between the two groups (*t*_25_ = 0.9, *p* > 0.05).

### Speech Recognition Measurements

The results of the SRT80 measurements are also summarized in Table 1. On average, the two groups of participants achieved very similar SRT80s, for both low-complexity (means: -1.9 vs. -2.1 dB SNR; *SD*: 0.8 vs. 1.0 dB SNR) and high-complexity (means: -1.2 vs. -1.3 dB SNR; *SD*: 1.1 vs. 0.7 dB SNR) sentences. Two independent *t*-tests showed no group differences for the two sentence types (both *t*_25_ < 0.2, both *p* > 0.05).

### Eye-Tracking Measurements

#### Response Times

On average, the eHA and iHA groups had response times of 1435 and 1749 ms (*SD*: 405 and 824 ms), respectively. To analyze these data further, we performed an analysis of variance (ANOVA) on the log-transformed data with participant group (eHA, iHA) as between-subject factor and linguistic complexity (low, high) as within-subject factor. We found no significant effects (all *p* > 0.05). Table 3 shows mean response times and SD for the two groups and levels of linguistic complexity.

**Table 3:** Means (and standard deviations) of the response times and processing times for the two levels of linguistic complexity (low, high) and participant groups (eHA, iHA).

		Response times (ms)	Processing times (ms)
Low	eHA	1432	833
		(466)	(215)
	iHA	1677	1165
		(783)	(483)
High	eHA	1438	1105
		(352)	(478)
	iHA	1822	1785
		(889)	(532)


#### Processing Times

On average, the eHA and iHA groups had processing times of 969 ms and 1475 ms (*SD*: 389 and 590 ms), respectively. Furthermore, both groups had longer (poorer) processing times for the high-complexity sentences (means: 1105 and 1785 ms; *SD*: 478 and 532 ms) than for the low-complexity sentences (means: 833 and 1165 ms; *SD*: 215 and 483 ms). To analyze these data further, we performed an ANOVA with participant group as between-subject factor and linguistic complexity (low, high) as within-subject factor. We found significant effects of participant group (*F*_(1,25)_ = 5.5, *p* < 0.026, $\eta_{p}^{2}$ = 0.18) and linguistic complexity (*F*_(1,25)_ = 21.0, *p* < 0.0001, $\eta_{p}^{2}$ = 0.46), but no interaction (*p* > 0.05). Table 3 shows mean processing times and SD for the two groups and levels of linguistic complexity.

### fMRI Measurements

**FIGURE 4:**
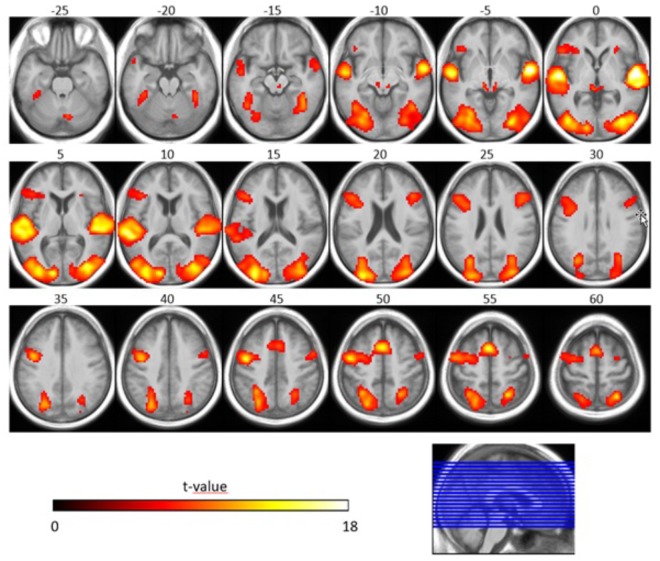
Speech-specific fMRI activation maps. The second-level *t*-statistic map for the SiN > noise contrast is thresholded at a significance level of *p* = 0.05, FWE-corrected (*t* = 6.02), with an extent-cluster threshold of 10 voxels, and is overlaid onto the group mean structural image. The numbers above the axial slices refer to their respective z-direction in MNI space (in mm), corresponding to the blue lines in the orthogonal view. Color-coding indicates the *t*-value.

Concerning the effect of stimulus type, we observed that the SiN stimuli led to more activation along bilateral superior temporal gyri (including primary auditory cortices), bilateral occipital gyri (including left superior occipital gyrus, bilateral middle occipital gyrus) and frontal lobe (including left superior frontal gyrus, left inferior frontal gyrus, right middle frontal gyrus, and left precentral gyrus) when compared to the noise-only stimuli (*T* = 6.02 *p* < 0.05, FWE-corrected, extended threshold *n* = 10). Figure 4 and Table 4 show brain regions with increased activation for the SiN > noise contrast.

**Table 4:** Maxima of brain regions for interaction effect of stimulus type (SiN > noise) for *p* < 0.05 FWE-corrected.

Brain region	Voxel level (T-val)	*X*	*Y*	*Z*	Cluster level (k-val)
**R superior temporal gyrus (TL)**	**24.60**	**60**	**-4**	**-3**	**895**
R superior temporal gyrus (TL)	17.24	60	**-**25	3	
**L superior temporal gyrus (TL)**	**18.35**	**-57**	**-10**	**0**	**1085**
L superior temporal gyrus (TL)	16.58	**-**60	**-**31	9	
L superior temporal gyrus (TL)	13.41	**-**48	**-**28	6	
**L superior occipital gyrus (OL)**	**15.76**	**-30**	**-91**	**18**	**2477**
L superior occipital gyrus (OL)	14.87	**-**15	**-**91	6	
L middle occipital gyrus (OL)	14.35	**-**42	**-**79	0	
**R middle occipital gyrus (OL)**	**15.75**	**36**	**-79**	**3**	**2075**
R superior parietal lobule (PL)	14.49	27	**-**58	57	
R middle occipital gyrus (OL)	13.85	33	**-**79	**-**6	
**L precentral gyrus (FL)**	**15.16**	**-45**	**-4**	**45**	**1499**
L supplementary motor area (FL)	14.87	0	11	54	
L precentral gyrus (FL)	11.90	**-**42	2	36	
**R inferior frontal gyrus (FL)**	**9.46**	**45**	**26**	**21**	**163**
R inferior frontal operculum (FL)	9.35	51	17	27	
**L inferior colliculus (BS)**	**9.31**	**-6**	**-31**	**-3**	**71**
R inferior colliculus (BS)	8.09	9	**-**28	**-**6	
**R precentral gyrus (FL)**	**8.70**	**54**	**5**	**42**	**101**
**R cerebellum declive**	**8.60**	**9**	**-70**	**-24**	**43**
**R insula (SL)**	**7.81**	**27**	**26**	**3**	**18**
**R middle frontal gyrus (FL)**	**7.06**	**30**	**-1**	**63**	**18**


*L, left; R, right; BS, brainstem; FL, frontal lobe; TL, temporal lobe; OL, occipital lobe; PL, parietal lobe; SL, sub lobar; CPL, cerebellum posterior lobe.*

**FIGURE 5:**
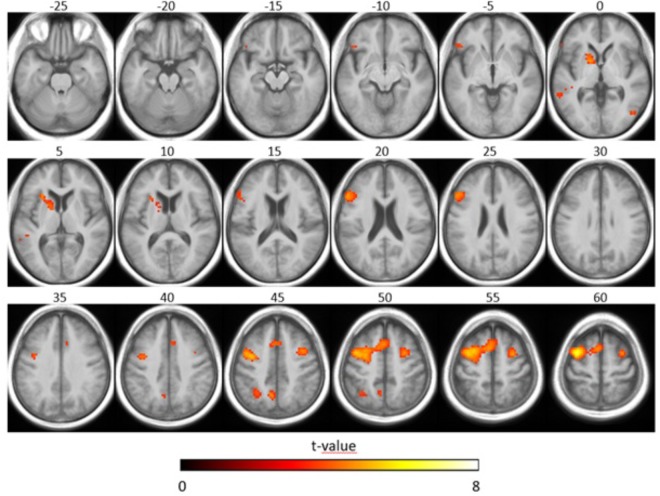
Functional magnetic resonance imaging activation related to linguistic complexity. The second-level *t*-statistic map for the SiN_high_ > SiN_low_ contrast is thresholded at a significance level of *p* = 0.001, uncorrected (*t* = 3.45), with an extent cluster threshold of 10 voxels, and is overlaid onto the group mean structural image. The axial slices shown are the same as in Figure 4. Color-coding indicates the *t*-value.

Concerning the effect of linguistic complexity, we observed that SiN_high_ stimuli led to more activation in bilateral frontal gyri (including left middle frontal gyrus, left inferior frontal gyrus, left precentral gyrus, left superior frontal gyrus, right middle frontal gyrus), left globus pallidus, left insula, left precuneus, right middle occipital gyrus, left middle temporal gyrus, and left inferior parietal lobule compared to the SiN_low_ stimuli (*T* = 3.45, *p* < 0.001, uncorrected, extended threshold *n* = 10). Figure 5 and Table 5 show brain regions with increased activation for the SiN_high_ > SiN_low_ contrast.

**Table 5:** Maxima of brain regions for interaction effect of linguistic complexity (SiN_high_ > SiN_low_) for *p* < 0.001 uncorrected.

Brain region	Voxel level (T-val)	*X*	*Y*	*Z*	Cluster level (k-val)
**L middle frontal gyrus (FL)**	**6.57**	**-33**	**5**	**63**	**666**
L precentral gyrus (FL)	5.63	**-**45	5	48	
L superior frontal gyrus (FL)	4.91	**-**3	11	57	
**L inferior frontal gyrus (FL)**	**4.85**	**-54**	**23**	**21**	**89**
**L globus pallidus**	**4.85**	**-15**	**5**	**0**	**87**
L insula	4.59	**-**27	20	6	
**R middle frontal gyrus (FL)**	**4.82**	**30**	**2**	**54**	**164**
R middle frontal gyrus (FL)	4.80	33	5	66	
R middle frontal gyrus (FL)	4.52	39	8	45	
**L precuneus (PL)**	**4.59**	**-9**	**-58**	**45**	**42**
**R middle occipital gyrus (OL)**	**4.17**	**48**	**-70**	**0**	**10**
L middle temporal gyrus (TL)	4.16	**-**45	**-**37	**3**	**32**
L middle temporal gyrus (TL)	4.04	**-**60	**-**43	3	
**L inferior parietal lobule (PL)**	**4.14**	**-30**	**-52**	**48**	**49**
**L inferior frontal gyrus (FL)**	**3.88**	**-54**	**29**	**-3**	**21**


*L, left; R, right; FL, frontal lobe; TL, temporal lobe; PL, parietal lobe, OL, occipital lobe.*

**FIGURE 6:**
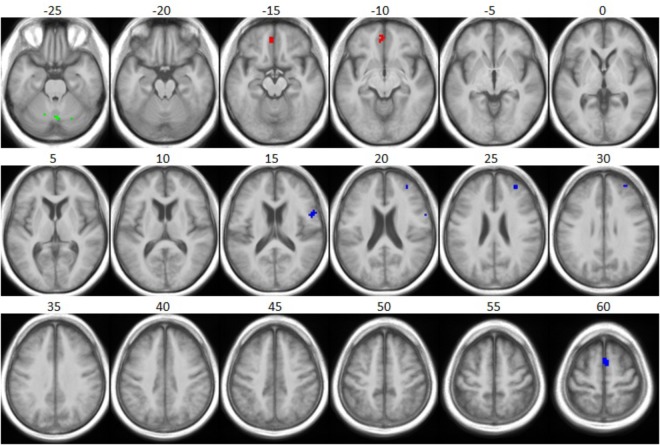
Group differences with respect to speech-specific and linguistic complexity-specific fMRI activation. The *t*-statistic maps for the respective interactions of group and stimulus condition, as obtained from two-sample *t*-tests, are thresholded at a significance level of *p* = 0.001, uncorrected (*t* = 3.47), with an extent cluster threshold of 10 voxels, and are overlaid onto the group mean structural image: iHA > eHA for SiN > noise (blue), iHA > eHA for SiN_high_ > SiN_low_ (green), and eHA > iHA for SiN_high_ > SiN_low_ (red). The contrast eHA > iHA for SiN > noise did not reveal any significant clusters. The axial slices shown are the same as in Figure 4.

Concerning the interaction between participant group and stimulus type, we observed that the iHA group showed more activation for the SiN > noise contrast in right frontal lobe (superior frontal gyrus, precentral gyrus, middle frontal gyrus) compared to the eHA group (*T* = 3.47, *p* < 0.001, uncorrected, extended threshold *n* = 10). Figure 6 (blue) and Table 6 show brain regions with increased activation for the iHA > eHA (SiN > noise) contrast. In contrast, the analysis did not reveal any voxels characterized by significantly stronger activation in the eHA group compared to the iHA group.

**Table 6:** Maxima of brain regions for the interaction effects of participant group x stimulus type (iHA > eHA for SiN > noise) and participant group × linguistic complexity (iHA > eHA for SiN_high_ > SiN_low_ and eHA > iHA for SiN_high_ > SiN_low_) for *p* < 0.001 uncorrected.

	Brain region	Voxel level (T-val)	*X*	*Y*	*Z*	Cluster level (k-val)
**iHA > eHA for SiN > noise**	R superior frontal gyrus (FL)	5.14	3	5	63	31
	R precentral gyrus (FL)	4.04	57	5	18	15
	R middle frontal gyrus (FL)	3.92	33	47	30	12
	R middle frontal gyrus (FL)	3.89	33	47	21	
**iHA > eHA for SiN_high_ > SiN_low_**	R cerebellum declive	4.19	3	-18	-24	11
	L cerebellum declive	4.17	-70	-67	-30	18
	R cerebellum declive	4.05	-24	-30	-30	11
**eHA > iHA for SiN_high_ > SiN_low_**	Medial orbitofrontal cortex (FL)	4.42	-3	47	-12	25


*R, right; FL, frontal lobe.*

Concerning the interaction between participant group and linguistic complexity, we observed that the iHA group showed more activation for the SiN_high_ > SiN_low_ contrast in bilateral cerebellum relative to the eHA group (*T* = 3.45, *p* < 0.001, uncorrected, extended threshold *n* = 10). Figure 6 (green) and Table 6 show brain regions with increased activation for the iHA > eHA (SiN_high_ > SiN_low_) contrast.

Concerning the interaction between participant group and linguistic complexity, we observed that the eHA group showed more activation for the SiN_high_ > SiN_low_ contrast in medial orbitofrontal cortex to the iHA group (*T* = 4.42, *p* < 0.001, uncorrected, extended threshold *n* = 10). Figure 6 (red) and Table 6 show brain regions with increased activation for the eHA > iHA (SiN_high_ > SiN_low_) contrast.

### Influence of HA Use Duration

It is conceivable that the effects of HA experience observed above increase with longer HA use duration. To explore this possibility, we entered HA use duration (in years) as a covariate or regressor in the analyses of the data from the eHA group. For the processing times, we found no significant influence of HA use duration. For the fMRI data, we entered the first-level contrast images for (1) the SiN > noise contrast and (2) the SiN_high_ > SiN_low_ contrast into separate linear regression analyses at the second-level, with HA use duration as a regressor. Applying a criterion threshold of *p* < 0.001 and a minimum cluster extent of five voxels, we found no effects of HA use duration for the SiN > noise contrast. For the SiN_high_ > SiN_low_ contrast, however, longer HA use duration was associated with increased activation in the medial occipital part of the lingual gyri as well as in the precuneus (i.e., the medial part of Brodmann area 7). The latter structure also showed more brain activation for sentences with high as opposed to low linguistic complexity (both groups) (see section “Results-fMRI Measurements”).

## Discussion

The purpose of the current study was to explore the influence of HA experience on sentence comprehension in noise and its underlying neural processes. Using the eye-tracking paradigm of Wendt et al. (2014), we measured processing times (i.e., the time taken to grasp the meaning of sentences presented against noise together with two pictures that either correctly or incorrectly depict the meaning of the sentences) based on eye-gaze measurements as well as behavioral response times (i.e., the time taken to identify the target picture via a button press after the spoken sentence). Additionally, we adapted this paradigm for fMRI measurements to obtain measures of brain activation, as inferred from BOLD contrasts. Our participants were groups of experienced and inexperienced HA users matched in terms of age, hearing loss, and working memory capacity. Consistent with previous findings, processing times increased with greater linguistic complexity and were also longer (poorer) for the iHA group than for the eHA group, despite comparable speech recognition performance. Also consistent with previous findings, the behavioral response times were not sensitive to these effects. Furthermore, we found some indications of stronger speech-specific activation in primarily frontal regions (i.e., insuperior frontal gyrus, right precentral frontal gyrus, and right middle frontal gyrus) of the iHA group. The observed group differences in brain activation were generally subtle and not entirely consistent with the research literature. Below, we discuss these findings in more detail.

### Effects of HA Experience and Linguistic Complexity on Processing Times

The effects of participant group and linguistic complexity on the processing times that we observed were consistent with the results of Wendt et al. (2015) and Habicht et al. (2016, 2018). In each of these studies, HA experience was related to shorter processing times and higher linguistic complexity to longer processing times. In view of the consistency of these findings, we conclude that untreated hearing loss leads to poorer SiN processing as measured via processing times and that HA experience can ameliorate these effects, at least if well-fitted HAs are used consistently for several months (see Habicht et al., 2018). In this context, it is worth noting that the response times do not appear sensitive to these effects. As argued in Habicht et al. (2016), this is likely a consequence of the ‘offline’ nature of this task. Offline tasks require the participant to respond *after* the stimulus presentation, for example by repeating words as in standard speech audiometry measurements. Thus, they cannot reveal when precisely speech comprehension occurred. ‘Online’ paradigms such as processing times, on the other hand, make it possible to investigate the influence of factors such as HA experience *during* the comprehension process. Presumably, they can therefore provide more detailed information about higher-level cognitive functions, including those affected by experience with amplified sound.

### Effects of Speech Processing on Cortical Activation Patterns

Our observation of an increased activation in a large-scale frontotemporal network (including bilateral superior temporal gyrus, precentral gyrus, inferior frontal gyrus) for SiN relative to noise-only stimuli is largely in line with other literature findings. Previous studies found stronger activation for SiN compared to noise-only stimuli in frontotemporal areas including bilateral temporal cortex (superior temporal sulcus, middle temporal gyrus), left inferior frontal gyrus and left precentral gyrus (e.g., Adank, 2012; Lee et al., 2016). These activated areas are believed to reflect the processing required to extract the meaning of speech and thus to achieve comprehension (Hickok and Poeppel, 2007; Peelle, 2012). The similarity between our results and those of previous studies lends support to the general validity of our experimental approach.

There were also signs of increased activation for high-complexity compared to low-complexity sentences. The additional activation was most evident in frontal areas (including bilateral middle frontal gyrus, left precentral gyrus, left inferior frontal gyrus, and left superior frontal gyrus) and temporal areas (including left middle temporal gyrus). In previous studies, these areas also showed more activation for complex compared to simple sentence structures (e.g., Peelle et al., 2004, 2011; Friederici et al., 2006). The study that can be compared most directly with ours is the one of Röder et al. (2002), in which stimuli with very similar (German) sentence structures were used. For a group of young normal-hearing participants, Röder et al. (2002) observed more activation in left inferior frontal gyrus for more complex compared to simpler sentences.

We also found more activation in left precuneus for high-complexity compared to low-complexity sentences. This finding seems to be in accordance with the results of Wilson et al. (2007) who presented auditory or audio-visual narratives to young normal-hearing listeners. Based on correlation analyses, these authors identified an extended network of brain regions involved in the processing of the narratives, which included the precuneus. Together, these results support the idea that the precuneus is involved in the comprehension of higher-level linguistic stimuli.

Lastly, we observed significantly more activation in occipital areas (including superior occipital gyrus and middle occipital gyrus) for SiN relative to noise-only stimuli and for high-complexity relative to low-complexity sentences. The occipital lobe is the visual processing center of the brain (e.g., Malach et al., 1995). To achieve sentence comprehension for SiN and high-complexity sentences, the participants probably looked more back and forth between the picture sets compared to noise-only and low-complexity sentences. To investigate brain activation due to eye movements, Zeki et al. (1991) presented a group of healthy participants with static or moving visual stimuli in an fMRI experiment. For the moving stimuli, they found more activation in the temporo-parieto-occipital junction, which they traced back to greater eye movements of the participants. Broadly speaking, this is in accordance with our finding of increased activation in occipital areas.

### Effects of HA Experience on Cortical Activation Patterns

Relative to the eHA group, the iHA group showed signs of more activation for the SiN > noise contrast in right frontal areas (including superior frontal gyrus, precentral frontal gyrus, and middle frontal gyrus). This suggests that HA acclimatization reduces the recruitment of frontal brain regions. Broadly speaking, this is in accordance with Erb and Obleser (2013) who observed that listeners with poorer hearing recruit additional frontal areas to compensate for speech processing deficits. However, we found no indications of more brain activation in temporal areas of the inexperienced users, as suggested by Peelle and Wingfield (2016).

Additionally, the iHA group showed signs of stronger activation related to higher linguistic complexity in the cerebellum. To our knowledge, the cerebellum has not been discussed yet in the context of hearing impairment. Nevertheless, previous studies have suggested that it may be involved in auditory and linguistic functions (e.g., Baumann et al., 2015; Schmahmann, 2016). In principle, the cerebellum could therefore also undergo brain changes as a result of untreated hearing loss. Further research would be required to investigate this issue.

In contrast to the iHA group, the eHA group showed signs of more sentence-specific activation in medial orbitofrontal cortex. To our knowledge, the orbitofrontal cortex has not been discussed in the context of hearing impairment yet either. Rich and Wallis (2016) showed that the orbitofrontal cortex is critically involved when a participant has to make a decision. The increased brain activation that we observed for the eHA group could, in principle, be a reflection of their better ability to identify the target picture, as apparent from their faster processing time measurements. Further research would also be required to investigate this issue.

At a more general level, it is worth speculating about the origin of the observed group differences in speech processing. The differences in acoustic stimulation that the two groups of participants were accustomed to from their daily lives probably manifested themselves in across-group differences in neural (re)organization. As a consequence, even if both groups followed the same hearing-related strategy when performing the different speech-in-noise tasks, the eHA group likely had an advantage because their auditory systems were adjusted for aided listening. It could also be that the two groups adopted different strategies, for example (not) focusing on (unfamiliar sounding) spectro-temporal cues that are only/more salient during aided listening. Our study cannot reveal which, if any or both, of these possibilities can explain the group differences that we found. We hypothesize that the effects of HA experience primarily manifest themselves at late (cortical) levels. Such a hypothesis would be consistent with the fact that the human brain is most malleable (and thus shaped by sensory experiences) in cortex. Further research needs to be devoted to this issue. In terms of potential implications for hearing rehabilitation, our study provides evidence for the importance of consistent HA use in relation to speech comprehension in noise, possibly related to less recruitment of brain regions outside the core sentence-comprehension network.

Lastly, it is important to point out that the group differences discussed above were subtle and based on a comparatively lenient thresholding criterion (see section “Materials and Methods-fMRI Measurements”). Because of the inherent complexity and inter-individual variability of cortical processes, differences in fMRI activation pertaining to group contrasts such as the ones tested here are, in general, likely to be small. To substantiate the initial findings from the current study, follow-up studies with larger sample sizes are needed for identifying and corroborating changes in cortical speech processing with HA use.

## Conclusion

In the current study, we used a cross-sectional design to explore the influence of HA experience on cognitive processes related to sentence comprehension in noise. To that end, we measured sentence processing times using an eye-tracking paradigm. Additionally, we performed fMRI measurements to explore brain activation patterns in response to SiN or noise-only stimuli. We found that inexperienced HA users had significantly longer processing times than participants with at least 1 year of bilateral HA experience. This is consistent with the findings of three earlier studies and implies poorer SiN processing due to untreated hearing loss, even when adequate speech audibility is ensured. Regarding the fMRI measurements, we found indications of stronger activation in several brain areas (especially in left frontal regions) for high-complexity compared to low-complexity sentences, consistent with the literature. Furthermore, the inexperienced HA users showed signs of more activation for SiN relative to noise-only stimuli in right frontal areas (including superior frontal gyrus, precentral gyrus, and middle frontal gyrus) compared to the experienced users. Because the observed group differences in brain activation were subtle, follow-up research is needed to investigate putative changes in cortical recruitment as a result of HA use in more detail.

## Ethics Statement

Ethical approval for all experimental procedures was obtained from the ethical review board of the University of Oldenburg. Prior to any data collection, all participants provided written informed consent in accordance with the Declaration of Helsinki. They were paid on an hourly basis for their participation.

## Author Contributions

JH formulated the research questions, designed, prepared and carried out the experiment, analyzed and interpreted the experimental data, and wrote the final manuscript. OB contributed to the design, preparation and execution of the fMRI measurements, the analysis and interpretation of the experimental fMRI data, and the writing of the final manuscript. BK contributed to the experimental design and the writing of the final manuscript. TN contributed to the formulation of the research questions, the experimental design, the analysis and interpretation of the experimental data, and wrote the final manuscript.

## Conflict of Interest Statement

The authors declare that the research was conducted in the absence of any commercial or financial relationships that could be construed as a potential conflict of interest.

## Footnotes

1 http://www.fil.ion.ucl.ac.uk/spm

2 That is, the averaged signal across voxels located in the cerebrospinal fluid/ventricles, as defined by an eroded mask obtained from individual probability maps created during the segmentation of the subject’s structural (T1-weighted) image.

3 This is referred to as ‘spike regression’ after Satterthwaite et al. (2013). For this purpose, we calculated DVARS [D referring to the temporal derivative of time courses, VARS referring to RMS variance over voxels as described in, for example, Power et al. (2012)], indexing the rate of change of the BOLD signal across the entire brain at each frame of data. Spikes were defined for each subject by DVARS values more than three standard deviations above the individual mean.

4 http://www.fmri.wfubmc.edu/download.htm
